# Polyvinyl Alcohol Carbazate as a Polymer-Based Antitumoral Agent

**DOI:** 10.3389/fonc.2020.598394

**Published:** 2021-01-11

**Authors:** Felix Sellberg, Robin Fröbom, Christian Binder, Erik Berglund, David Berglund

**Affiliations:** ^1^ Section of Clinical Immunology, Department of Immunology, Genetics and Pathology, Uppsala University, Uppsala, Sweden; ^2^ Section of Endocrine and Sarcoma Surgery, Department of Molecular Medicine and Surgery, Karolinska Institute, Stockholm, Sweden; ^3^ Department of Clinical Science, Intervention and Technology (CLINTEC), Division of Transplantation Surgery, Karolinska Institute and Karolinska University Hospital, Stockholm, Sweden

**Keywords:** antitumoral polymer, immune therapy, melanoma, T cell infiltration, melanoma *in vivo* model, drug discovery

## Abstract

Development of treatment resistance is a major concern during treatment of cancer, and there is an unmet need for therapeutic strategies with novel modes of action. Polyvinyl alcohol carbazate (PVAC) is a polymer compound with unique biological properties. Herein, we describe the antitumoral effects of PVAC. Three well-established cell lines GIST-T1, B16.F10, and A375 were used to determine the *in vitro* antitumoral effects of PVAC. Assessments included light microscopy, cell viability, cell cycle, and apoptosis assays. *In vivo* treatment safety and efficacy were characterized in one immunocompetent (B16.F10) mouse model and one athymic nude (MDA-MB-231) mouse model. Excised tumors were measured, weighed, stained for Ki-67, CD3, and histopathologically evaluated. Intact PVAC expressed a non-linear dose-response antitumoral effect *in vitro*, whereas its separate components, PVA and carbazate, did not display antitumoral effects alone. *In vivo*, PVAC induced a significant intratumoral CD3^+^ T-cell recruitment in immunocompetent mice (B16.F10), which was associated with tumor growth inhibition. Although growth inhibition was not significant in athymic mice (MDA-MB-231), histopathological evaluation detected an increase in stromal tissue and leukocyte infiltration. In conclusion, we present evidence for PVAC antitumoral effects both *in vitro* and *in vivo*. The mode of action was not elucidated *in vitro*, but a potential mechanism of *in vivo* activity was observed, characterized by an increase of immune cells into both immunocompetent and athymic mice. This finding warrants further study to validate its possible role as an immunomodulatory polymeric agent.

## Introduction

Cancer is one of the leading causes of death worldwide with an estimated 10 million deaths per year (1). Despite the development of new drugs, leading to improved patient survival across several cancer types, long-lasting effects are burdened by intrinsic and acquired drug resistance. There is thus a continuous unmet need for alternative treatment strategies with novel modes of action.

Polymers constitute a class of chemical compounds that can be modified in several ways. Recently, polymer technologies have been used to enhance antitumoral treatments. These applications include using polymers as a drug-delivery system, and integration to the backbone of cytostatic drugs to modify the pharmacokinetic properties (2, 3) and more recently, as direct antitumoral agents (4–7). These polymers have been designed to interact with cancer cell membrane due to their net negatively charged membranes (8). Modifications of polymers also allow for different sized molecules, which leads to an enhanced permeability and retention effect (EPR). This leads to an increased extravascular polymer accumulation because of diffusion through permeable and defect endothelial walls of rapidly forming malignant blood vessels (9–11). A size-dependent accumulation of polyvinyl alcohol (PVA) in tumor tissue has been described in mice (12). PVA was used as a backbone in a molecule initially developed to be drug conjugated, but pilot studies suggested that the polymer possessed direct antitumoral activity, which was confirmed in a neuroblastoma and melanoma model (4).

Carbazate-activated polyvinyl alcohol (PVAC) consists of a PVA backbone, functionalized with carbazate moieties that molecularly target biological electrophiles such as carbonyls, and PVAC is capable of binding multiple carbonyls. Carbazate contains a neutrophilic nitrogen atom with similar properties to an amine. Compared to an amine, carbazate allows for increased stabilization of electrons making it more reactive and the formed bonds more stable. Normally, the excess of such substances is limited by reducing agents such as glutathione, or enzymes such as aldehyde dehydrogenase (13).

Initially, PVAC was designed as a crosslinker of hyaluronic acid to form a cartilage-like hydrogel (14). However, initial results when working with cell lines in relation to the compound yielded unexpected effects on cell morphology and viability. This spurred us to further investigate the substance in a tumor system. Herein, we use an experimental model of different cancer cell lines, which we combine with mouse models with different immune status, one immunocompetent B16.F10 melanoma model, and an athymic breast cancer MDA-MB-231 model, to investigate the antitumoral effects and the safety of intratumoral PVAC injections.

## Materials and Methods

### Compounds

Polyvinyl alcohol carbazate (PVAC) is a functionalized version of PVA (n) with multiple pendant carbazate groups (m) (15). The level of substation is approximately 10%. The size of the PVA backbone is on average 18 kDa (13,000–23,000 g/mol) and PVAC has a molecular weight between 15 and 35 kDa. PVAC used for the experiments was synthesized at Ångström Laboratory at Uppsala University or by specific polymers (Castries, France). PVAC was stored freeze-dried at −20°C and dissolved in desired medium or Milli-Q® water (Millipore, MA, USA) prior to the different experiments. PVAC is a polymeric substance derived from a PVA backbone modified with carbazate groups at some hydroxyl moieties. Ethyl carbazate (EC, a low-molecular-weight carbazate compound) and PVA (at the same size used in PVAC synthesis) were used as controls. EC and PVA were dissolved in deionized water yielding stock concentration of 50 mg/ml. Both compounds were purchased from Sigma (Stockholm, Sweden).

### Cell Lines

GIST-T1 is a human gastrointestinal stromal tumor (GIST) cell line, purchased from Cosmo Bio Co. Ltd. (Tokyo, Japan). B16.F10 (murine) and A375 (human) are both melanoma cell lines. A375 was chosen due to its prevalence in the literature and similar transcriptional profile to melanoma biopsies (16). A375 was purchased from ATCC (Manassas, Virginia, USA) while B16.F10 was a kind gift from Adlego AB (Stockholm, Sweden). MDA-MB-231 (breast cancer) was used in a mouse model by Adlego AB. Cells were maintained in DMEM medium supplemented with 10% FBS, 1% penicillin/streptomycin. In addition, GIST-T1 medium was supplemented with 1% GlutaMAX. Cells were cultured in an incubator, at 37°C and 5% CO_2_. GIST-T1 was used for CellTiter-Glo assay and flow cytometry, while B16.F10 and A375 were used for flow cytometry experiments.

### Cell Viability Assay

0.25×10^4^–1×10^4^ GIST-T1 cells were seeded into each well in a 96-well plate and incubated overnight to allow adherence. PVAC, EC, and PVA were then added into each well or vehicle (Milli-Q water) with subsequent incubation for 24 or 48 h. CellTiter-Glo assay (Promega, MI, USA) is an ATP-based luminometric method that was performed according to protocol. In brief, cells were removed from incubator to allow adjustment to room temperature after which 100 µl of room temperature CellTiter-Glo reagent was added to each well. The plates were put on a shaker for 5–10 min. Cells rested for 5–10 min before measurement on an EnVision 2103 Multilabel Reader (Perkin Elmer, MA, USA).

### Flow Cytometry

Medium was prepared with the addition of PVAC in six different concentrations with dilutions of a factor of 5 (2.5 to 0.8 µg/ml). GIST-T1 and B16.F10 cells were incubated for 48 h, with the outlined concentrations. A375 cells were assessed at 24, 48, 72, and 96 h, in a dose or wash protocol. After 48 h media was discarded, cells were washed in PBS twice and then new media was added, containing PVAC (dose) or not (wash), the culture was then continued for another 48 h. Wells were harvested daily, and cells were analyzed by flow cytometry for apoptosis/necrosis assay [Annexin V + 7-AAD or PI (GIST-T1)] and cell cycle analysis (using PI in A375 cell line, **Supplementary Figure B**).

For apoptosis flow cytometry cells were harvested and washed twice in PBS, resuspended in Annexin Binding Buffer (10 mM HEPES, 150 mM NaCl, 2.5 mM CaCl2 in PBS), and transferred to glass flow cytometry tubes to which 5 µl of Annexin V FITC (Sigma, MI, USA) and 10 µl of 7-AAD (Sigma, MI, USA) or propidium iodide (PI) (Thermo Fisher, MA, USA) was added. The samples were incubated for 20 min prior to analysis. Samples were analyzed on an Accuri C6 (B16.F10) or FACSverse flow cytometer (A375 and GIST-T1) (BD, Franklin Lakes, NJ, USA). All samples were exported as.FSC files and processed in FlowJo (FlowJo LCC, Ashland, OR, USA). Gating strategies and representative examples can be seen in **Supplementary Figure A**. For cell cycle analysis a built-in tool in FlowJo was used with the Watson (Pragmatic) univariate model. In parallel to flow cytometry experiment, images of cells in culture were also taken each day with Samsung A3 cellular device (Seoul, South Korea).

### *In Vivo* Model of Melanoma and Breast Cancer

This part of the study was performed by Adlego AB, at Astrid Fagreaus Laboratory (Stockholm, Sweden), in two parts. The first part encompassed 36 immunocompetent C57BL/6J mice injected with B16.F10 cells. The second part encompassed 36 athymic nude mice injected with MDA-MB-231 tumor cells. The dosages were chosen from *in vitro* experiments, with one low dose (0.5 mg/ml) and one high dose (10 mg/ml). Sodium chloride (NaCl) was used as a vehicle. The high dose was also chosen to evaluate safety of the compound.

#### Melanoma Model

To establish the tumor 10^6^ B16.F10 cells were injected in the right rear flank. After injection into the C57BL/6J mice the mice were monitored twice weekly recording weight, general health, and tumor size. When the tumor size had reached at least 0.05 cm^3^ mice were stratified into three groups of 12 mice per group based on tumor size. All three groups of C57BL/6J mice were subjected to intra/peri-tumoral injections three times per week at a volume of 100 µl per injection for the duration of the study period. One group served as a control group and received NaCl (vehicle) injections; the two treatment groups received different doses of PVAC, 0.5 and 10 mg/ml, respectively. Tumor size and health status were recorded three times weekly during the treatment period. Mice were euthanized when average tumor size of the control group reached 2 cm^3^ in volume. Tumors were then excised, weighed, and divided into two parts. One part was frozen in isopentane on dry ice and stored at −80°C the second part was placed in 4% buffered formaldehyde until shipment to Micromorph AB (Lund, Sweden) for immunohistochemical analyses.

#### Breast Cancer Model

To establish the tumor 1.5 × 10^6^ MDA-MB-231 cells were injected into the fat surrounding the mammary gland of athymic nude mice [Crl:NU(NCr)-FOXn1^nu^]. The mice were monitored twice weekly recording weight, general health, and tumor size. When the average tumor size was about 0.1 cm^3^ mice were stratified into three groups of 12 mice per group based on tumor size. All three groups were subjected to 28 days of twice-weekly intra/peri-tumoral injections at a volume of 100 µl per injection. One group served as a control group and received NaCl (vehicle) injections. The remaining two groups received different doses of PVAC, as described above. The mice were euthanized on day 29 and tumors were excised, weighed, and divided into two parts handled as described above.

### Immunohistochemistry and Histology

Sectioning and staining of tumors were carried out by Micromorph AB. All sections were dehydrated, cleared, and infiltrated with paraffin automatically in a TISSUE-TEK V.I.P (Miles Scientific, Newark, DE, USA). Sections were acquired from peripheral and central parts of tumors. All tumors were stained with hematoxylin eosin (HE). Sections from B16.F10 were also stained with anti-CD3 and anti-KI67 (**Table 3**). HE staining was carried out with Mayer’s hematoxylin (Bio-Optica, Milano, Italy) for 6 min followed by washing in running water for 10 min. Then sections were stained in eosin (Bio-Optica) for 3 min, washed in distilled water, and finally dehydrated and mounted.

Slides used for immunohistochemistry were first deparaffinated before antigen retrieval by boiling in boric acid (pH 8.0) + 0.2% triton-X 100 for 20 min. Slides were cooled at room temperature for 20 min and then washed in PBS. They were incubated with primary antibodies for 1 h (**Table 1**) in PBS with 5% goat serum (Jackson ImmunoResearch, Cambridgeshire, UK), followed by washing three times in PBS. Secondary antibody was added (**Table 3**) and slides were incubated for 30 min, followed by washing three times in tris buffer and incubation in 3,3-diaminobenzidine for 5 min. Finally, they were rinsed in distilled water and counter stained in hematoxylin for 5 s before dehydration and mounting.

**Table 1:** Antibodies used for immunohistochemistry.

Target	Type	Source
Anti-CD3	Rabbit monoclonal	Abcam, ab16669
Anti-KI67	Rabbit monoclonal	Abcam, ab16667
Secondary antibody	BrightVision anti-rabbit/HRP	Immunologic

A senior pathologist scored all tumor slides in a blinded fashion using a Leica DMRX microscope. B16.F10 was scored with regard to tumor morphology, number of CD3^+^ cells, and Ki-67 expression. The scoring of CD3^+^ cells and Ki-67 expression was: 0 (no positive cells); very few positive cells (0–1+); few positive cells (1+); few to moderate numbers of positive cells (1–2+); moderate number of positive cell (2+); moderate to high number of positive cells 2–3+); high numbers of positive cells (3+); MDA-MB-231 was scored with regard to tumor morphology and leukocyte infiltration.

### Gene Expression Analysis

Tumors from the *in vivo* models were snap frozen in liquid nitrogen and stored in a liquid phase nitrogen freezer until further analysis. Tumors were dissected and digested using QIAshredder (Qiagen, Hilden, Germany) columns for homogenization followed by RNeasy mini kit (Qiagen) spin columns. Total RNA was subjected to quality control with Agilent Tapestation according to the manufacturer’s instructions. To construct libraries suitable for Illumina sequencing, the Illumina TruSeq Stranded mRNA sample preparation protocol was used with starting concentration between 0.1 and 4 µg total RNA. The protocol includes mRNA isolation, cDNA synthesis, ligation of adapters, and amplification of indexed libraries. The yield and quality of the amplified libraries was analyzed using Qubit by Thermo Fisher and quality was checked by using Agilent Tapestation. The indexed cDNA libraries were normalized and combined and the pools were sequenced on the Illumina NextSeq 550 for a 75-cycle v2 sequencing run, generating 75 base pair, single read with dual index. Base calling and demultiplexing was done using Illumina bcl2fastq (v2.20.0) software. Readings were aligned to Ensemble reference genome GRCh38 and GRCm38, respectively, using STAR (v2.5.2b). Quality control was done using FastQC (v0.11.8) and MultiQC (v1.7). Gene assignment was done using featureCounts (v1.5.1). Sample group comparisons were done using R package DESeq2 (v1.22.2). Sample group comparisons were done in DESeq2 (v1.22.2), generating fold changes, Wald test p values, and p-values adjusted for multiple testing (Benjamini and Hochberg method).

### Hydrophobicity and Hydrophilicity Assay

To study the hydrophobic and hydrophilic interactions of PVA and PVAC, quartz crystal microbalance with dissipation (QCM-D) was used. Gold sensors (Biolin Scientific, Sweden) were coated with 1-undecanethiol (Sigma) in a self-assembling monolayer, to form a hydrophobic surface and uncoated gold sensors were used as a hydrophilic surface. They were then washed in type-1 water and dried with N2 gas. The sensors were then placed in a QSense Pro (Biolin Scientific), the experiment was initiated with PBS being passed over the sensors for 5 min followed by PBS containing 2.5 mg/ml of PVAC or PVA for 45 min and finally 10 min of PBS. Mass shift (ng/cm^2^) and dissipation was recorded. Two hydrophilic and hydrophobic sensors were run in two experiments resulting in 4 observations per surface.

### Statistics

Figure data is presented as averages ± standard deviation (SD), significant differences (p value < 0.05) are denoted with *. Statistics in text are reported as p-value, mean difference between groups tested, 95% CI (when possible to calculate), in the format of, *p = 0.05*, z% (x–y), where z is the mean difference between the groups and x and y are the bounds of the 95% CI. Multiple group analyses were carried out using one parameter one-way ANOVA for parametric data (e.g., tumor mass) and Kruskal-Wallis for non-parametric data (e.g., lymphocyte invasion). Two-way ANOVA was used to analyze two parameter data sets (e.g., tumor size over time). ANOVA was followed with multiple comparisons testing between the experimental groups and the control group using Tukey´s in the case of one-way ANOVA and Dunnett’s in the case of two-way ANOVA. When possible, technical replicates were nested in the analysis; when this was not possible the mean from the replicates was used instead. When comparing categorical variables such as abscess formation between groups, Fisher’s test was used.

### Ethical Permits

*In vivo* studies with B16.F10 and MDA-MB-231 were conducted with permit N37/15 by the regional animal experimental ethics committee in Stockholm North.

## Results

### Polyvinyl Alcohol Carbazate, Polyvinyl Alcohol, and Ethyl Carbazate Effect on Cell Viability

**Figure 1:**
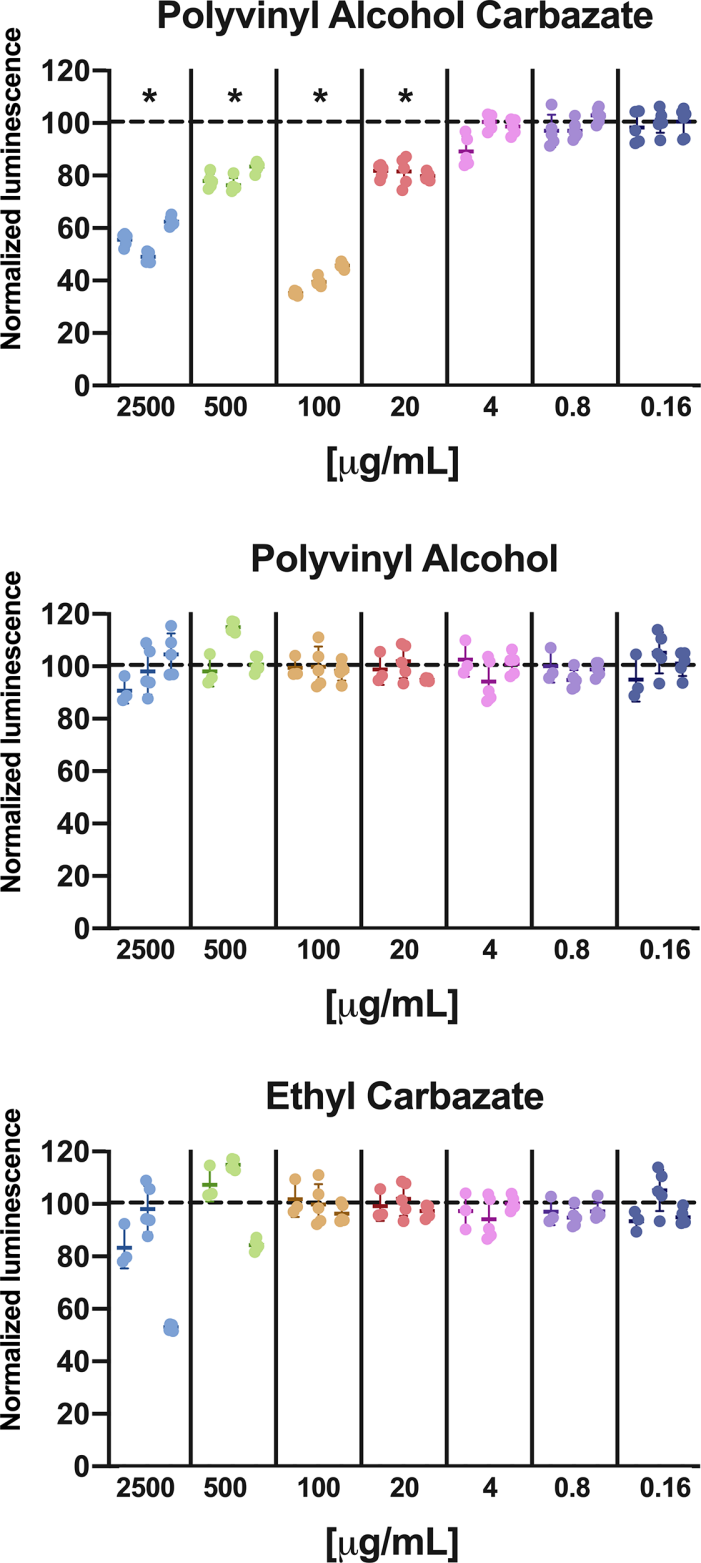
Cell viability in GIST-T1 cells treated with PVAC, ethyl-carbazate, or polyvinyl alcohol at different concentration. The results are normalized against vehicle (dotted line) after 48 h. PVAC reduced the ATP content, indicating loss of viable cells. The dose-relationship was non-linear, with dose optimum of about 100 µg/ml (mean 59.9%, CI 95% 49.4–70.4) and at 2,500 µg/ml (44.3%, CI 95% 33.8–54.8) compared with 500 µg/ml (mean 20.8%, CI 95% 10.3–31.3) and 20 µg/ml (mean 19.0%, CI 95% 8.5–29.4). Polyvinyl alcohol (PVA) and EC did not significantly affect ATP content. n = 3 (biological replicates). * denotes p-value < 0.05.

Addition of PVAC to cell culture led to reduced ATP in the wells reflecting a reduced number of viable cells. The effect did not follow a linear response curve; instead a peak concentration was observed of about 100 µg/ml. At concentrations below 20 µg/ml no reduction in viable cells was seen (**Figure 1**).

To assess the potential cytotoxicity from the chemical constituents of PVAC, the backbone PVA at the same size as the synthesized PVAC and a low-molecular-weight carbazate, ethyl carbazate (EC) was used at different concentrations. None of the compounds decreased cell viability (**Figure 1**), indicating that the combination of the carbazate group with a large backbone is needed to observe the effects of PVAC. The molar ratio between PVA and carbazate is about 5:1; therefore a concentration of 2,500 µg/ml PVAC corresponds to 500 µg/ml carbazate.

**Figure 2:**
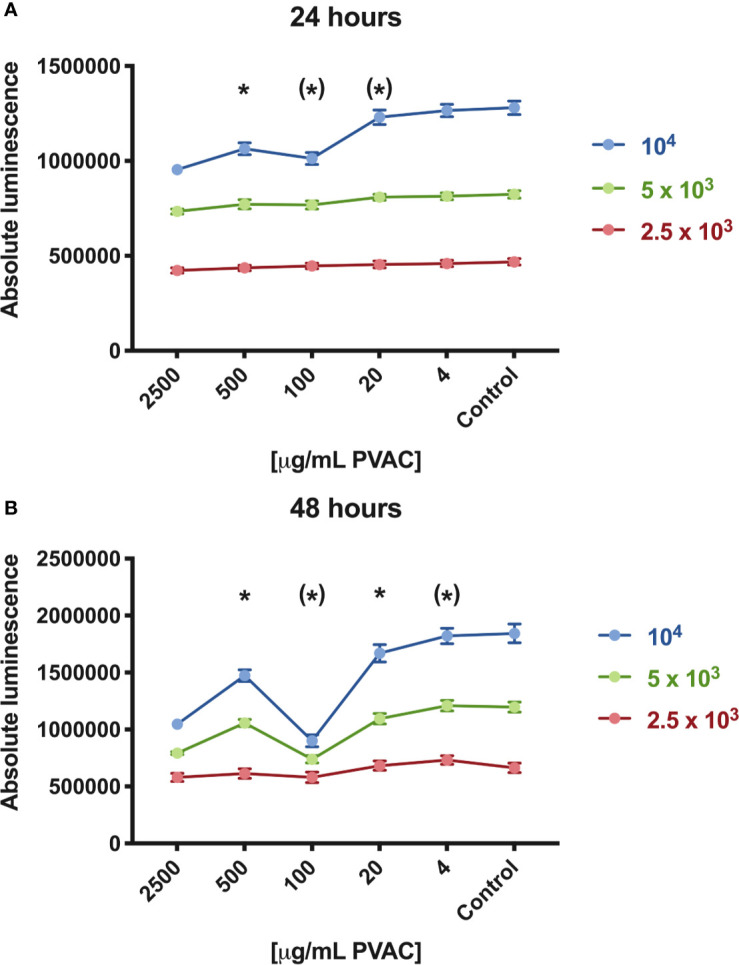
Cell viability of GIST-T1 cells cultured in the presence of polyvinyl alcohol carbazate (PVAC) at varying seeding densities [2,500 (low), 5,000 (medium), and 10,000 (high) cells/well, respectively]. **(A)** At 24 h concentrations of 100–2,500 µg/ml reduced viability in high seeding density, whereas in 2,500 µg/ml in medium seeding density. **(B)** After 48 h 20–2,500 µg/ml reduced viability in high seeding density, and 2,500 and 100 µg/ml in medium seeding density. No significant decrease in cell viability was observed in low seeding density. n = 3 (biological replicates). * denotes p-value < 0.05.

Cells were seeded at different cell densities, showing that increased density of cells led to a decrease in cell viability. The effect was most pronounced with high seeding density (10^4^ cells/well) and time (48 h), indicating that the cell viability reduction upon PVAC treatment depends on both time and seeding density (**Figure 2**). The non-linear dose-response relationship was observed in high seeding density at both time intervals, while only at 24 h for medium density (5×10^3^ cells/well). The use of absolute luminescence also shows increased values between 24 and 48 h, indicative of viable and proliferating cells.

#### Flow Cytometric Evaluation of Cell Apoptosis/Necrosis Using Annexin V/7-AAD or Propidium Iodide

Flow cytometric evaluation using Annexin V/7-AAD or PI was used to examine apoptotic and necrotic cells. A375 human melanoma cells were used in an extended protocol, to assess the possibility to rescue cells from PVAC treatment. GIST-T1 cells and B16.F10 cells were assessed after 48 h. After 48 h, most cells (A375, B16, and GIST-T1) significantly increased in late apoptotic/necrotic cells by PVAC concentrations ranging from 20 to 500 µg/ml. The effect was not significant for other concentrations.

**Figure 3:**
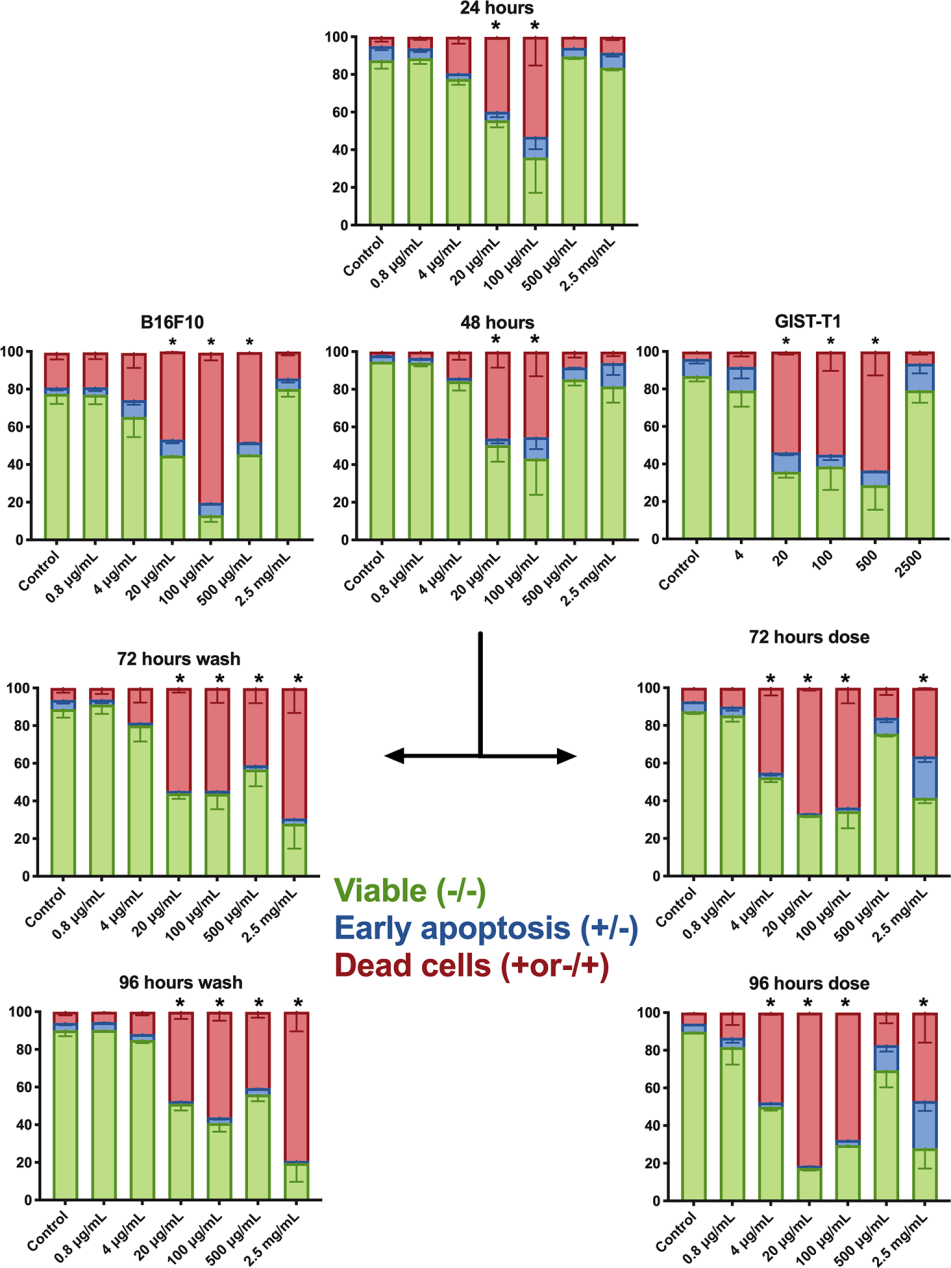
Flow cytometry annexin V/7-AAD (A375 and B.16.F10) or propidium iodide (PI) assay (GIST-T1). This depicts the distribution of cells within respective population at different concentrations; each graph represents a different time point. At 48 h, all three cell lines are shown: B16.F10, A375, and GIST-T1, the rest of the data presented in the figure is from culture with A375. After 48 h, a dose or wash protocol was done. Bars denoted with * had significantly fewer viable cells (double negative) and an increased cell population of 7-AAD^+^ cells. The cells receiving a second dose of polyvinyl alcohol carbazate (PVAC) at a concentration of 2.5 mg/ml also had an increased number of early apoptotic cells (Annexin V^+^/7-AAD^−^). For representative examples of flow cytometry and gating, see **Supplementary Figure A**. D, dose; W, wash. n = 3 (biological replicates). * denotes p-value < 0.05.

To assess the possibility of rescuing cells by removing PVAC from the culture media, a protocol was designed were cells were either dosed with new PVAC or washed and then cultured in media not containing PVAC. The culture was then continued for another 48 h. Concentrations 20–100 µg/ml displayed an increase in the number of dead cells and a decrease in viable cells throughout the culture. The highest concentration 2,500 µg/ml displayed an increased number of dead cells and a decrease in viable cells after 72 and 96 h in culture regardless of washing or dosing. However, when using 500 µg/ml changes were only noted following washing. After dosing the highest concentration (2,500 µg/ml) as well as a lower concentration (4 µg/ml) there were an increased number of dead cells and a decrease in viable cells after 72 and 96 h in culture (**Figure 5**). No recovery occurred when PVAC was washed from culture. A non-linear dose response was evident in both viability assays, morphology, and with flow cytometry (**Figures 2**, **6**). This effect did not seem to be cell line specific (**Figure 3**).

### Morphological Evaluation of Cells in Culture Exposed to Polyvinyl Alcohol Carbazate or Vehicle

**Figure 4:**
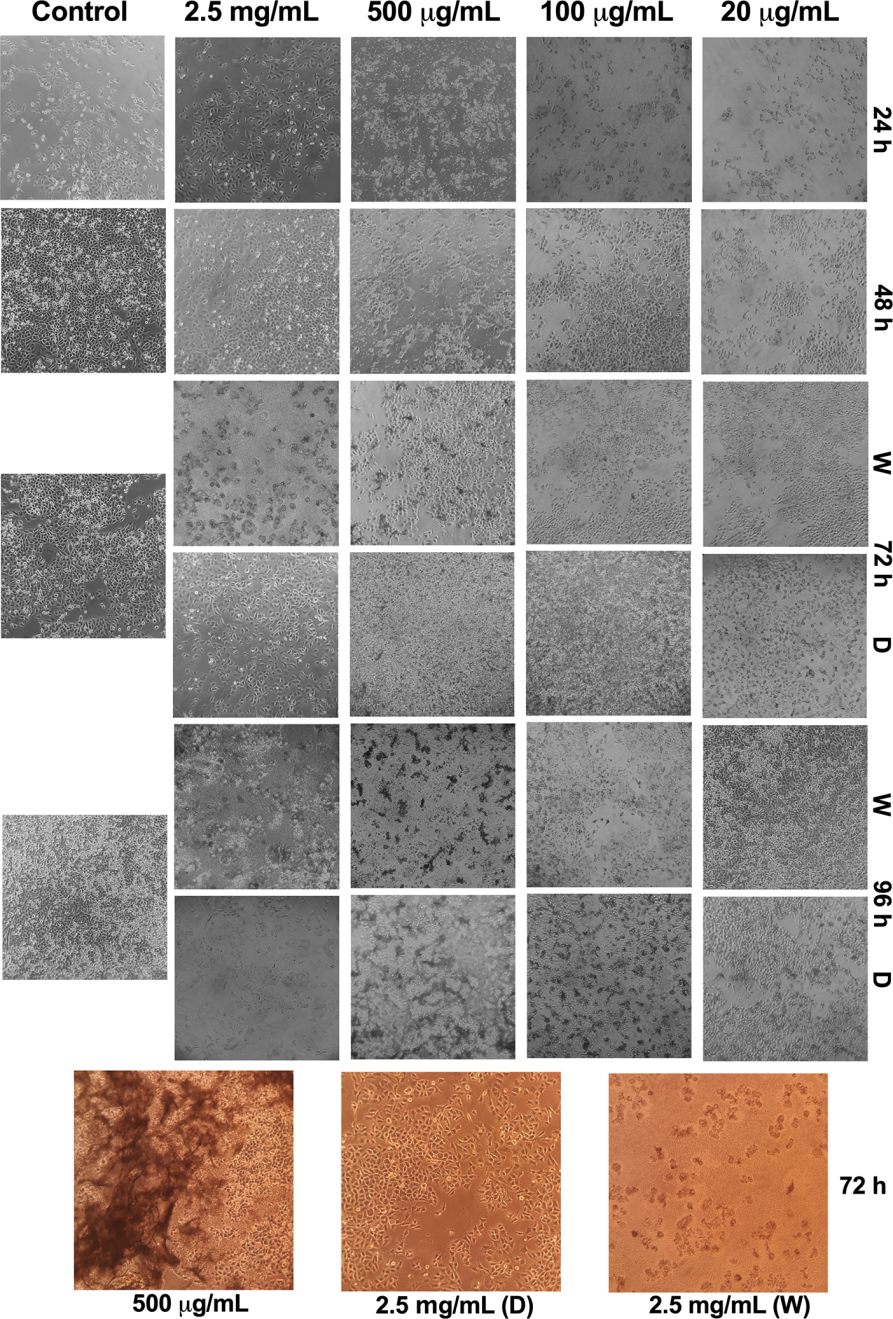
Representative images of morphological appearance of A375 cells exposed to polyvinyl alcohol carbazate (PVAC) at different concentrations. Rows represent time points (h) and columns represent concentrations of PVAC. Concentrations lower than 20 µg/ml had a similar morphological appearance as the control, and were therefore excluded. Black and white images are the same passage as the representative example for flow cytometry. Bottom three pictures in color are from different cell passage. D, dose; W, wash.

The control confluence increases rapidly during culture with cells growing initially in a monolayer, followed by multiple layer growth. Culture visual changes can be observed during the first day after exposure to PVAC (**Figure 4**). Confluency and morphology appear affected in the cells with 20 and 100 µg/ml PVAC. In addition, cells exposed to 500 µg/ml PVAC also display apoptotic bodies morphologically. After 48 h in culture the effects are similar with increased confluency. After washout the cells exposed to 2,500 µg/ml collapses into an unrecognizable mass of necrosis, 500 µg/ml also appears more affected after washout while 20 and 100 µg/ml slowly recover in confluency and morphology. After dosing 2,500 µg/ml cells keep their gross morphology initially but after 96 h in culture cells appear deformed and few in number. Moreover, 20–500 µg/ml appear increasingly affected over time with large masses of cellular debris. The colored pictures display the same phenomenon as observed previously in 2,500 µg/ml with the cells in the washout group collapsing and the dosed cells with intact morphology. The final left-most picture depicts crosslinking of cellular debris into large sheets, potentially a direct effect by PVAC. This is mainly evident at 100 and 500 µg/ml but does not occur at lower or higher concentrations. Because the solubility of PVAC is affected when more carbazate is bound another possible explanation is that we observe PVAC precipitating (**Figure 4**). The PVAC treated and control cells on the cell cycle did not differ during the culture, except for the highest dose (2,500 µg/ml), **Supplementary Figure B**.

### *In Vivo* B16.F10 and MDA-MB-231

**Figure 5:**
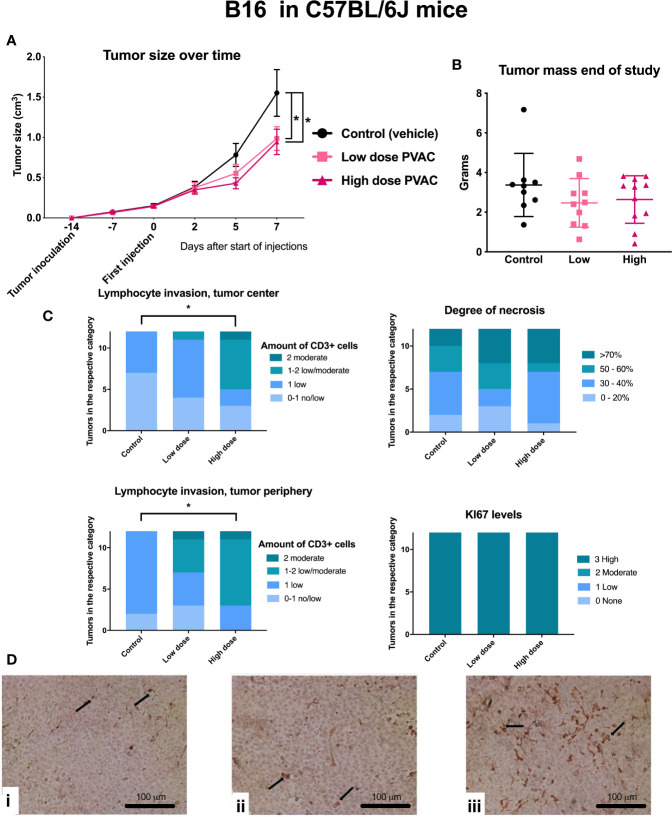
C57BL/6J mice were inoculated with B16.F10 tumor cells and subsequently intratumorally treated with polyvinyl alcohol carbazate (PVAC) (either low dose 0.5 mg/ml or high dose 10 mg/ml) or vehicle. **(A)** Injections performed on days 0, 2, 5, and 7. Five days after the first injection, tumor size was smaller in the high dose PVAC group compared to control (p < 0.05 mean 0.34 cm^3^, CI 95% 0.01–0.69). On day 7 the tumor size was smaller in both low dose and high dose PVAC groups (p < 0.05 mean 0.6 cm^3^ CI 95% 0.27–0.95 and p < 0.05 mean 0.57 cm^3^ CI 95% 0.22–0.92 respectively). **(B)** Shows tumor mass at the end of the study after tumor excision. Tumor mass showed a reduction trend. **(C)** Scoring of histological features, CD3 and Ki-67 staining. The number of CD3^+^ cells were higher in the high dose PVAC group compared to the control group both in the tumor center (p < 0.05 mean 1.42 rank difference CI 95% 1.09–1.74 *vs*. 2.42 CI 95% 1.78–3.05) and periphery (p < 0.05 mean 1.83 rank difference CI 95% 1.59–2.08 *vs*. 2.47 CI 95% 2.47–3.2). **(D)** displays histology of tumors stained with CD3: **i** control (vehicle), **ii** low dose PVAC, **iii** high dose PVAC.

In B16.F10 six mice that died before the end of the study were excluded from statistical analysis due to incomplete data (**Table 2**). PVAC at both dosing levels led to a reduced tumor size over time (**Figure 5A**), whereas mass did not differ after excision of the tumors (**Figure 5B**). In the high dose group, an increased number of CD3^+^ cells were observed both in the tumor center and periphery (**Figure 5C**). The amount of Ki67+ cells was high in all the groups which is to be expected as B16.F10 is a rapidly proliferating cell line, in addition the degree of necrosis is high as the growth of the tumor does not allow for expansion of supporting tissues. The histopathological evaluation revealed an increased number of CD3^+^ positive cells intratumorally following PVAC treatment (**Figure 5D**).

**Table 2:** B16.F10 melanoma model characteristics, including tumor size and mass.

B16.F10 C57BL/6J	Control (vehicle)	Low dose (0.5 mg/ml)	High dose (10 mg/ml)
Mice per group	12	12	12
Mice deceased before study end	3	2	1
Tumor size end of study (cm^3^)	1.55 ± 1.00	0.99 ± 0.49	0.95 ± 0.55
Excised tumor mass (g)	3.37 ± 1.59	2.47 ± 1.22	2.64 ± 1.20

**Figure 6:**
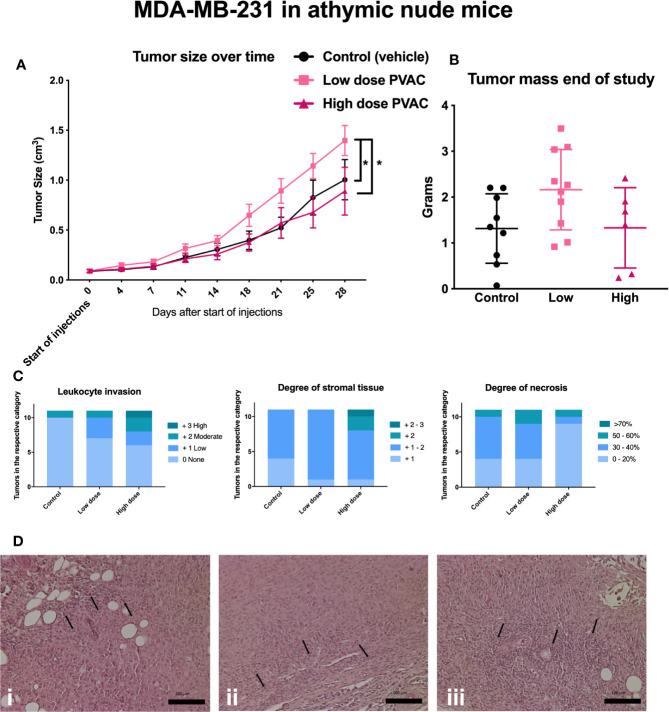
Athymic nude mice were inoculated with MDA-MB-231 tumor cells and subjected to intratumoral polyvinyl alcohol carbazate (PVAC) (high or low dose) or vehicle injections. **(A)** Injections over time, each tick represents new injection. After 21, 25, and 28 days the tumor volume was significantly higher in the PVAC low dose group when compared to the control and the high dose group (at 28 days p < 0.05 mean 0.39 cm^3^ CI 95% 0.13–0.66 and p < 0.05 mean 0.51 cm^3^ CI 95% 0.21–0.8, respectively). No other differences between the groups were noted. **(B)** shows tumor mass at the end of the study. No significant difference between the groups was noted. **(C)** Scored leukocyte infiltration, stromal tissue and necrosis; no significant differences were noted. **(D)** Shows histological slides of tumors, **i** control (vehicle), **ii** low dose PVAC, **iii** high dose PVAC. Arrows indicate leukocytes.

In MDA-MB-231, three mice failed to develop established tumors and were excluded prior to treatment administration. Three mice, one from each group, were euthanized due to a tumor volume exceeding two cm^3^. In total, five mice were euthanized due to infections, of which four were from the PVAC high dose group (**Table 3**). PVAC at a low dose led to an increased tumor size over time (**Figure 6A**), no other differences were noted between the groups at any timepoints; mass did not differ after excision of the tumors (**Figure 6B**). Groups did not differ in the histological parameters, including stromal content, degree of necrosis, and leukocyte invasion (**Figure 6C**). Comparing the degree of necrosis to the B16.F10 tumors it is relatively lower in the MDA-MB-231, this is due to the slower growth rate of the tumor. The histopathological evaluation reported signs of therapeutic effect, described as increased leukocyte count and increased stromal tissue in the high dose PVAC group (**Figure 6D**). A number of cases of abscess formation was noted in the MDA-MB-231 mouse model, although not significantly different between the groups, and it being a common complication in the model, it is something to keep in mind for future studies.

**Table 3:** MDA-MB-231 breast cancer model characteristics, including tumor size and mass.

MDA-MB-231	Control (vehicle)	Low dose (0.5 mg/ml)	High dose (10 mg/ml)
Mice per group	11	11	11
Euthanized due to abscess*	1	0	4
Euthanized due to volume > 2 cm^3^	1	1	1
Tumor size end of study (cm^3^)	1.00 ± 0.60	1.40 ± 0.47	0.89 ± 0.59
Excised tumor mass (g)	1.32 ± 0.76	2.16 ± 0.88	1.33 ± 0.88

*no significant differences between the groups.

### Gene Expression Analysis From Tumors in *In Vivo* Experimentation

**Figure 7:**
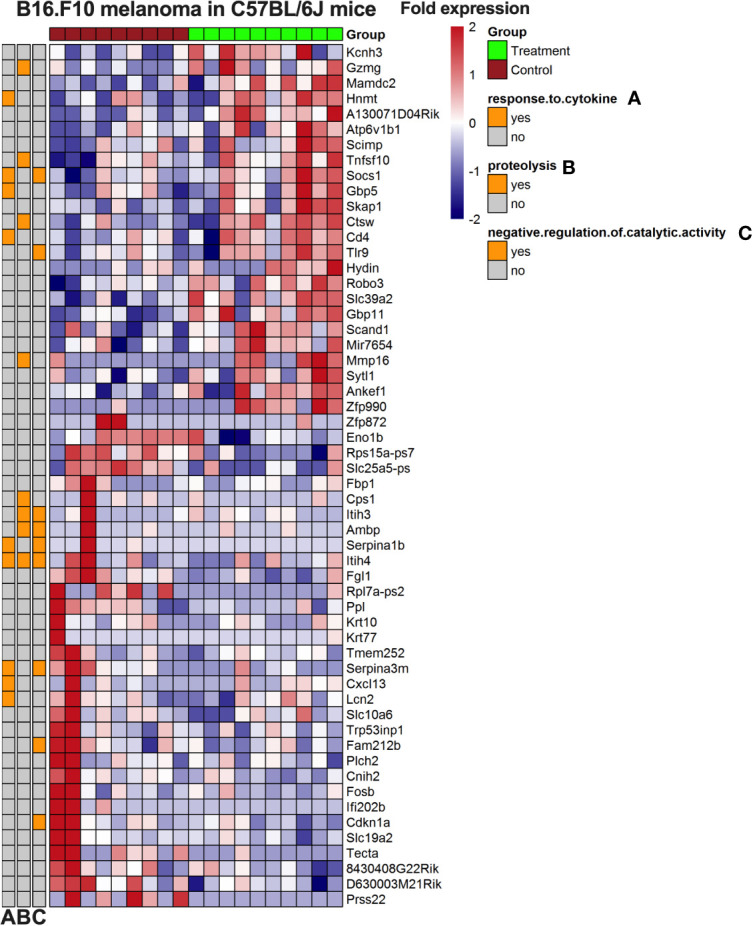
Heatmap of gene expression from B16.F10 melanoma *in vivo* experiment. Tumors from the polyvinyl alcohol carbazate (PVAC) high and control group were analyzed. The genes are listed to the right of the heatmap. The scale is fold change with 2-fold increased as red and 2-fold decreased as blue. Clustering was done and associated gene ontology (GO)-terms investigated.

We first analyzed the RNAseq data using adjusted p values, where no significant altered gene expression signatures could be identified (data not shown) and there was no significant clustering. We therefore used a lowered threshold for detecting significant gene changes (fold change > 1.5; unadjusted p-value 0.005). Using this approach with GO enrichment three pathways were identified: response to cytokines, proteolysis, and negative regulation of catalytic activity (**Figure 7**). In this gene set, we found several genes involved in the immune system and inflammation, such as the upregulation of CD4, TLR9, CTSW, SKAP1, and GBP1. Taken together, these results suggest a possible role for gene expression signatures that is different between control and PVAC-treated tumors although these genes were explored with more liberal cut offs to allow GO enrichment analysis. These data need further validation to confirm the role of the individual genes.

#### Polymer Characterization Using Quartz Crystal Microbalance With Dissipation

The mass shift from PBS was negligible for both surfaces (4.0 ± 7.8 ng/cm^2^). On the hydrophobic sensors the mass shift was low for both PVAC (31.8 ± 4.5 ng/cm^2^) and PVA (13.8 ± 13.6 ng/cm^2^). On the hydrophilic sensors the mass shift was rapid and high for both PVAC (179.2 ± 28.6 ng/cm^2^) and PVA (262.7 ± 52.9 ng/cm^2^) recorded 1 min after start of circulation with solutions containing the polymers. The final recording, after 10 min of washout was significantly lower for PVAC (198.8 ± 22.6 ng/cm^2^) compared with PVA (414.2 ± 6.8 ng/cm^2^). Dissipation was also lower for PVAC (1.2 ± 0.0) compared to PVA (2.1 ± 0.0). In combination, this indicates that the PVA was able to form a tighter layer as more molecules were packed on the surface; this reorientation was also a slower progress because the mass density on the sensor increased 60% over the time PVA containing solution circulated after the initial occupation.

## Discussion

Further to being used as an adjunct with conventional chemotherapy agents, polymers have been shown to have direct antitumoral effects, with encouraging results from studies specifically targeting tumor cells with non-toxic effect on normal cells. The antitumoral effect have been shown in chemotherapy-resistant cells (7) as well as dormant cancer cells (6), both of which represent major clinical problems in the treatment of cancer. Based on observed *in vitro* PVAC effects we set out to determine whether similar *in vivo* effects could be established.

The chemical constituents of PVAC, carbazate, and PVA did not induce a response *in vitro*. PVA is known to be non-toxic and has been shown to facilitate hematopoietic stem cell expansion *in vitro* (17). The non-linear dose relationship (**Figure 1**) was unexpected and consistent across all cell lines tested. An increased portion of apoptotic cells have been shown in lower doses of polymer-based compounds compared to higher doses (18). We hypothesize that this could be explained by the multiple binding sites on PVAC. A similar dose-response can be seen with antibodies containing multiple binding sites, which is at least in part explained by a *prozone* or *hook effect*, meaning that certain reactions do not appear when antibody is either too high or too low in relation to target. In a similar manner a certain number of sites on PVAC may need to interact with cells to elicit a response. Another factor supporting this is that carbazate alone did not induce a response because the reactivity of the carbazate group is the same but multiple sites are lacking. We also show that the treatment effect depends on treatment time, concentration, and cellular seeding density. A higher seeding density led to a more pronounced anti-tumoral effect. It is more common that a low seeding density is associated with increased cytotoxicity, in part because of the relatively higher concentration of the compound. Other factors might be that low seeding density cells displays increased proliferation and therefore are more susceptible to chemotherapeutic agents (19–21). PVAC as a macromolecule could act differently compared to conventional antitumoral compounds. PVAC might interact with adherent cells as a form of extracellular matrix, where confluency is determined not only by the absolute number of cells, but also by cell interaction with the PVAC molecule.

Because other polymer-based agents have used a membrane disrupting mode of action, we used a QCM-D approach to study whether PVAC was able to interact with cell membranes as previously described polymers; however, we found that PVAC was more hydrophilic than hydrophobic. For other polymers, it was found that when hydrophobic groups decreased in mole-percentage, the antitumoral effect also decreased (6). Therefore, we believe that PVAC does not act through a membrane disruption mechanism similar to that described for other polymers (6, 7). In addition, PVAC have been shown to decrease hemolysis in red blood cells (RBC) and stabilize the RBC membrane without interacting with the membrane directly (15). However, it is known that cancer produces excess of reactive carbonyl species (RCS) in its lipid layer (22, 23) and RCS are known targets of PVAC. By targeting such reactive species, it could be speculated that PVAC may interact with the membrane of cancer cells.

Immunotherapy has recently shown itself as a successful treatment modality within modern oncology. Tumor cells possess the ability to suppress immune cells through several mechanisms (24). To overcome the intratumoral tolerance, checkpoint inhibitors (CPIs) work by inhibiting suppressive signals, allowing T cells to become activated (25), which have been found successful in the treatment of malignant melanoma (26). In our stringent melanoma mouse model, we observed an increased T cell and leukocyte infiltration into the tumor, accompanied by a significant tumor growth inhibition. In clinical samples, tumor-infiltrating lymphocytes have been shown to be a predictive marker for response to CPI treatment (27). Although cytotoxic T cell infiltration is generally seen as a positive indicator, increased numbers of regulatory T cells have been associated with a poor outcome in patients (28). In addition, we performed RNA sequencing of the tumor samples identifying genes involved in immune system and inflammatory response which were upregulated; these results are preliminary and need to be further validated. Although no significant tumor growth inhibition was found in athymic breast cancer mouse model, an increased rate of leukocyte infiltration intratumorally was observed, together with changed histological architecture, including increased stromal tissue, indicative of a therapeutic effect. The lack of a response in the athymic breast cancer model could be explained by the lack of a competent adaptive immune system because increased T cell infiltration was observed in the melanoma model. Melanoma has been a model system for immunotherapeutic treatment strategies, and its immunogenicity is likely attributed to the high number of neoantigen generated from a high mutational burden. Breast cancer shows a lower number of neo-antigens and a diminished response to immunotherapy (29, 30).

Complete remission is difficult to achieve in an established melanoma model and combination therapies are often employed in clinical oncology. Although PVAC monotherapy did not result in complete remission, the treatment effect is encouraging and warrants further investigation. Today, several therapeutic agents provide meaningful clinical benefit for patients although complete remission was not observed in preclinical models (31, 32). In addition, combination treatments have been shown to increase the efficacy further, notably combining different therapeutic approaches (33).

To summarize, polymers have been found to be useful tools because they can be designed for a wide variety of properties. In this study, we used a carbazate-activated polyvinyl alcohol to determine its antitumoral effects that was characterized *in vitro* by decreased cell viability and increased late apoptotic/necrotic cells that could not be observed with either component of PVAC alone. Findings from an *in vivo* melanoma model showed inhibited tumor growth and increased T cell infiltration, reflecting possible immunomodulatory capability of PVAC. The intratumoral treatment was well tolerated, with no obvious toxicity observed. This finding warrants further studies to validate its possible role as an immunomodulatory polymeric agent.

## Data Availability Statement

The datasets presented in this study can be found in online repositories. The names of the repository/repositories and accession number(s) can be found in the article/**Supplementary Material**.

## Ethics Statement

The animal study was reviewed and approved by permit N37/15 by the regional animal experimental ethics committee in Stockholm North.

## Author Contributions

Conceptualization: FS, RF, DB, EB. Methodology: FS, RF, CB. Validation: RF. Formal analysis: FS, DB. Investigation: FS, RF. Resources: DB, EB. Data curation: FS, RF. Writing—original draft preparation: FS, RF. Writing—review and editing: FS, RF, CB, DB, EB. Visualization: FS, RF, CB. Supervision: DB, EB. Project administration: DB, EB. Funding acquisition: DB, EB. All authors contributed to the article and approved the submitted version.

## Conflict of Interest

EB, DB, and FS have applied for patent protection for the use of PVAC as an antitumoral agent.

The remaining authors declare that the research was conducted in the absence of any commercial or financial relationships that could be construed as a potential conflict of interest.
