# Supplementary material for: Polyvinyl Alcohol Carbazate as a Polymer-Based Antitumoral Agent
Source: Front Oncol. 2021 Jan 11;10:598394. doi: 10.3389/fonc.2020.598394 (PMC7831155; doi:10.3389/fonc.2020.598394)
Supplement: Supplementary file 1 [file DataSheet_1.docx]

Supplementary Material

# Supplementary Data A Gating Strategies


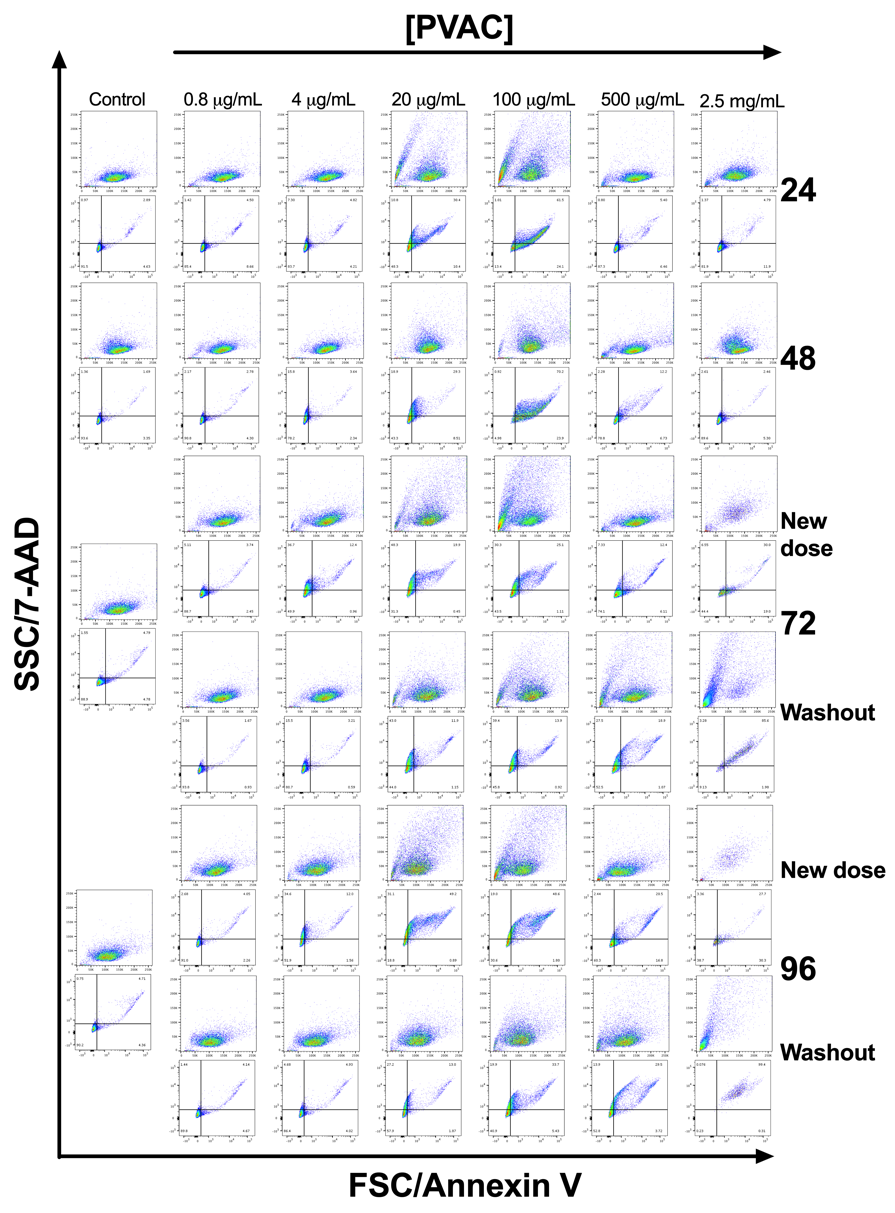


**Figure A.** Gating strategies for Annexin V/7-AAD assay analysis. As supplement to Figure 5 and 6.

# Supplementary Data B cell cycle analysis

## Methods

For cell cycle flow cytometry samples were washed x 2 in PBS and then resuspended in 500 µL of PBS, the entire volume of PBS was then added to 4.5 mL of ice cold 70% EtOH and the samples were then fixated for a minimum of 2 h (all samples were analyzed within 48 h) on ice or at 4°C. Samples were then centrifuged at 500g x 5 min and supernatant EtOH discarded, cells were resuspended in 5 mL of PBS and centrifuged at 500g x 5 min and supernatant PBS discarded. Finally, cells were resuspended in 1 mL of PI staining buffer containing 10 µg/mL of PI (Sigma) and 100 µg/mL of RNase (Sigma, Stockholm, Sweden) and transferred to glass flow cytometry tubes, samples were incubated for 30 min prior to analysis and analyzed within 60 min.

_
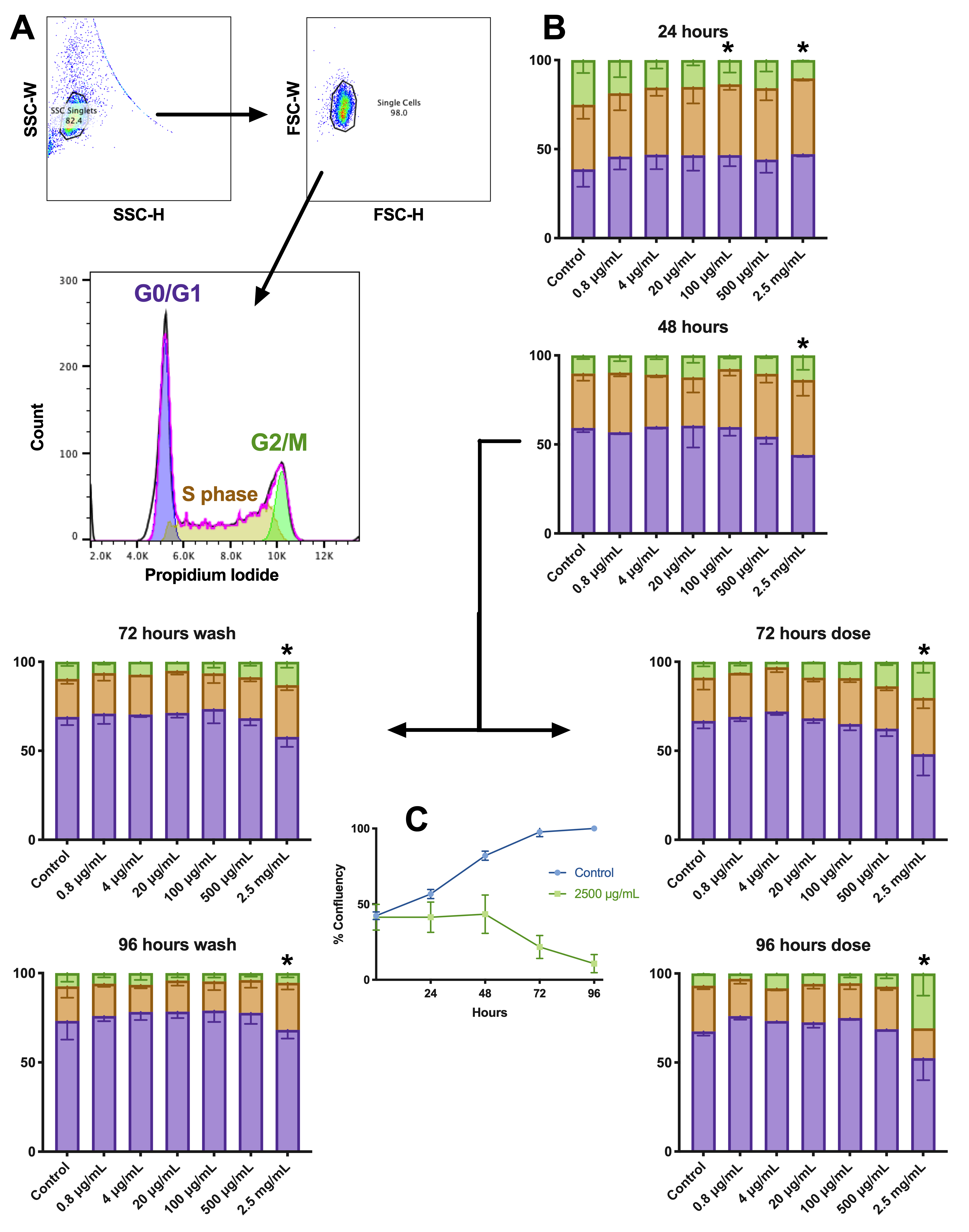
_

**Figure B.** Flow Cytometry cell cycle analysis assay from cell culture of A375 human melanoma, three cultures from different passages in triplicates. **A** Representative of example of gating strategy, single cells were identified using SSC W/H and FSC W/H gates and then analyzed with FlowJo cell cycle analysis tool. **B** Bars represent different treatments and graphs represent timepoints. Distribution of phases in cell cycle represented with different colors. **C** Confluency over time in culture as judged from visual inspection in the control and 2.5 mg/mL. * denotes p-value < 0.05.

# Data availability repository information

All data generated in the present work apart from genomic data is present at a public repository at Mendeley data, DOI: 10.17632/rk6hc2dz4c.1

All genomic data is available at ENA (EMBL-BI) the study accession is PRJEB40401, for individual data files please see the table below for accession.

| Accession | Data file |
| --- | --- |
| ERR4757641 | FASTQ from B16.F10 experiment |
| ERR4757642 | FASTQ from B16.F10 experiment |
| ERR4757643 | FASTQ from B16.F10 experiment |
| ERR4757644 | FASTQ from B16.F10 experiment |
| ERR4757645 | FASTQ from B16.F10 experiment |
| ERR4757646 | FASTQ from B16.F10 experiment |
| ERR4757647 | FASTQ from B16.F10 experiment |
| ERR4757648 | FASTQ from B16.F10 experiment |
| ERR4757649 | FASTQ from B16.F10 experiment |
| ERR4757650 | FASTQ from B16.F10 experiment |
| ERR4757651 | FASTQ from B16.F10 experiment |
| ERR4757652 | FASTQ from B16.F10 experiment |
| ERR4757653 | FASTQ from B16.F10 experiment |
| ERR4757654 | FASTQ from B16.F10 experiment |
| ERR4757655 | FASTQ from B16.F10 experiment |
| ERR4757656 | FASTQ from B16.F10 experiment |
| ERR4757657 | FASTQ from B16.F10 experiment |
| ERR4757658 | FASTQ from B16.F10 experiment |
| ERR4757659 | FASTQ from B16.F10 experiment |
| ERR4757660 | FASTQ from MDA-MB-231 experiment |
| ERR4757661 | FASTQ from MDA-MB-231 experiment |
| ERR4757662 | FASTQ from MDA-MB-231 experiment |
| ERR4757663 | FASTQ from MDA-MB-231 experiment |
| ERR4757664 | FASTQ from MDA-MB-231 experiment |
| ERR4757665 | FASTQ from MDA-MB-231 experiment |
| ERR4757666 | FASTQ from MDA-MB-231 experiment |
| ERR4757667 | FASTQ from MDA-MB-231 experiment |
| ERR4757668 | FASTQ from MDA-MB-231 experiment |
| ERR4757669 | FASTQ from MDA-MB-231 experiment |
